# Diversity of Silica-Scaled Chrysophytes of Two Tropical Islands: Phu Quoc and Con Son (Viet Nam)

**DOI:** 10.3390/life12101611

**Published:** 2022-10-15

**Authors:** Evgeniy Gusev, Nikita Martynenko, Dmitry Kapustin, Hai Doan-Nhu, Lam Nguyen-Ngoc

**Affiliations:** 1 A.N. Severtsov Institute of Ecology and Evolution, Russian Academy of Sciences, Leninsky Prospect 33, 119071 Moscow, Russia; 2 Joint Russian-Vietnamese Tropical Research and Technological Centre, 63 Nguyen Van Huyen, Nghia Do, Cau Giay, Hanoi 11307, Vietnam; 3 K.A. Timiryazev Institute of Plant Physiology, Russian Academy of Sciences, Botanical Street 35, 127276 Moscow, Russia; 4 Institute of Oceanography, Viet Nam Academy of Science and Technology, 01 Cau Da, Vinh Nguyen, Nha Trang 650000, Vietnam

**Keywords:** Viet Nam, tropical islands, flora, silica-scaled chrysophytes, *Mallomonas*, *Synura*, *Chrysosphaerella*, *Spiniferomonas*, *Lepidochromonas*

## Abstract

The present paper focuses on the silica-scaled chrysophytes from two tropical islands: Con Son (Con Dao Archipelago) and Phu Quoc, located in Viet Nam. Electron microscopy revealed fifty-seven taxa, of which forty-one belong to the genus *Mallomonas*. The others are species of the genera *Synura* (5), *Paraphysomonas* (8), *Spiniferomonas* (1), *Chrysosphaerella* (1) and *Lepidochromonas* (1). This is the first report of the genus *Lepidochromonas* from Viet Nam. In addition, two species from the genus *Paraphysomonas* are reported for the first time in the country. Six taxa from the genus *Mallomonas* and five taxa from the genus *Paraphysomonas* were not identifiable to the lower rank and may represent new species for science. The overall diversity of the silica-scaled chrysophytes of the islands is very high. A number of rare taxa endemic to Southeast Asia were found.

## 1. Introduction

Chrysophytes are unicellular or colonial algae characterized by heterokont flagella, chloroplasts with chlorophyll *a* and *c* and endogenous silicified stomatocysts, and they include about 1200 species in about 112 genera [1]. To date, a consistent system of morphological criteria for distinguishing taxa has only been developed for a small number of chrysophytes (orders Synurales and Paraphysomonadales and some representatives of Chromulinales and Ochromonadales). This morphological system is consistent with the available molecular genetic information on these taxa [2,3,4,5,6,7]. Although sexual reproduction is known among chrysophytes [8,9,10,11], the biological species concept is difficult to apply to this group, and the morphological species concept, which is based on the presence or absence of distinct taxonomic features, remains the most useful. For instance, in *Mallomonas*, species can be distinguished by differences in scale and bristle structure; in *Synura*, the taxa differ in the structure of the spine, keel, basal pore diameter, presence/absence of meshes, labyrinthic pattern, papillae, etc. of scales [12]; in *Paraphysomonas* sensu stricto, the most useful features are the shape and dimensions of the scale base and spine, the presence or absence of a rim and annulus and the shape of the spine tip [3]; in *Chrysosphaerella*, the species differ in the structure of plate-scales and/or spine-scales.

Many species of scale-bearing chrysophytes, including endemic taxa, were found during investigations of some tropical Asian countries including Malaysia [13,14], India [15,16], Sri-Lanka [17], Singapore and Indonesia [18], tropical and subtropical regions of China [19,20] as well as tropical areas in Africa [21,22,23,24] and South and Central America [25,26,27,28,29,30,31,32]. However, there are still considerable gaps in our knowledge of the species diversity, taxonomy, ecology and geographic distribution of tropical freshwater algae [33,34]. Our previous investigations have revealed a very rich flora of silica-scaled chrysophytes from Viet Nam [35,36,37,38,39,40,41,42]. These studies have revealed 83 taxa from the genera *Mallomonas*, *Synura*, *Chrysosphaerella*, *Spiniferomonas* and *Paraphysomonas*. Thirteen unidentified morphotypes of *Mallomonas* scales and two of *Synura* have also been reported in freshwaters of the country, but all of these studies were carried out on the mainland, while the numerous islands of Viet Nam have not been explored.

The aim of this paper is to study the silica-scaled chrysophytes in different freshwater habitats located on two tropical islands in Viet Nam.

## 2. Materials and Methods

**Figure 1:**
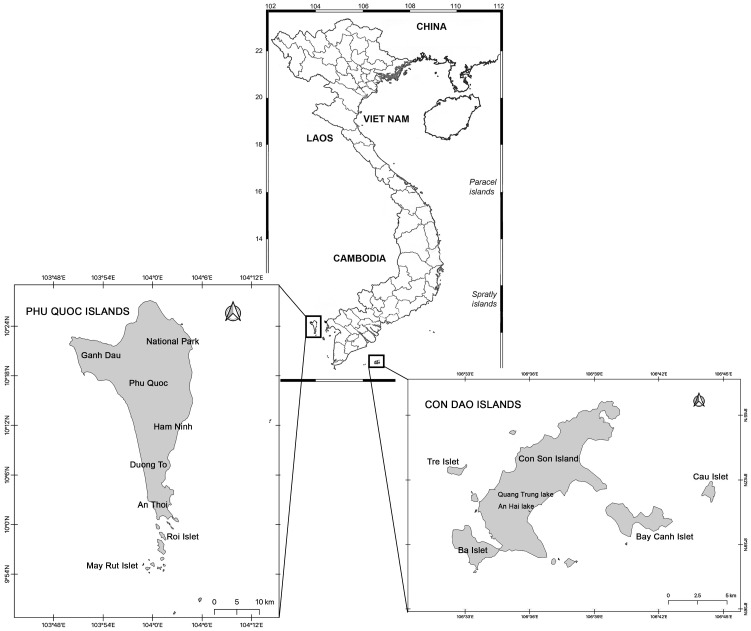
Geographical position of the studied area.

Samples from Phú Quốc Island, Kiên Giang Province and Côn Sơn Island, Bà Rịa–Vũng Tàu Province, Viet Nam, are included in this study (Figure 1). Information about these areas can be found in our previous works [43,44,45]. We studied seven localities on Phú Quốc Island in April 2015, including the upper stream and surrounding swamp of Duong Dong River, Duong Dong Reservoir, two ponds adjacent to the reservoir, swamps near the estuary of the Cua Can River and two small water bodies in the northwest part of the Island (Table 1, Figure 1). Phú Quốc lies off the Cambodian coast, to the south of Kampot and 40 km (22 nmi) west of Hà Tiên, the nearest coastal town in Viet Nam. Roughly triangular in shape, the island is 50 km (31 mi) long from north to south and 25 km (16 mi) from east to west at its widest. It is also located 17 nautical miles (31 km) from Kampot, 62 nautical miles (115 km) from Rạch Giá and nearly 290 nautical miles (540 km) from Laem Chabang, Thailand. Phú Quốc has both a terrestrial national park and a marine protected area. Phu Quoc National Park was established in 2001 as an upgrade of a former conservation zone. The park covers 336.57 km^2^ (129.95 sq mi) of the northern part of the island.

We studied seven localities on Côn Sơn Island (Côn Đảo Archipelago) in 2015, including Anh Hai Lake, two water bodies on the site of a dry Quang Trung Reservoir and four aquaculture ponds. Con Son, located in Côn Đảo District (Bà Rịa–Vũng Tàu Province), is an island of the southeast geography zone located in the South China Sea. The geographic coordinates of the district are from 106°31′ to 106°45′ east longitude and from 8°34′ to 8°49′ north latitude. Con Dao is situated far from Vung Tau city (179 km). Con Dao is located close to the equator and enjoys a warm tropical climate. The rainy season runs from May through September. From November to February, the island is subjected to high winds. The average temperatures during the year are 27 °C, reaching a high of 33 °C. Many of the islands were given protected status in 1984 as part of Côn Đảo National Park. This natural preserve was subsequently enlarged in 1998. The endangered species protected within the park include the hawksbill turtle, the green turtle, dolphins and the dugong. Ecosystems represented in the park include seagrass meadows, mangroves and coral reefs. Terrestrial landscapes and freshwaters are also under protection in Côn Đảo National Park.

Samples were collected from the surface water layer using a plankton net (mesh size = 20 μm) and from sediments. For studies with the scanning electron microscope (SEM), an aliquot of each sample was washed by repeated centrifugation in deionized water. Drops of the washed sample were dried or digested for 4–5 min in sulfuric acid with potassium dichromate. The samples were placed on aluminum stubs and coated with gold for 10 min. Observations were carried out with a JEOL 6510 LV or LEO-1420 SEM. For studies with the transmission electron microscope (TEM), formvar-coated grids (EMS FF200-Cu-50, Electron Microscopy Sciences, Hatfield, PA, USA) were used, and observations were made on a JEM-1011 TEM. Specific conductance, pH and temperature measurements were performed using a Hanna HI 9828 device (Hanna Instruments Inc., Smithfield, RI, USA). Samples were taken during the expedition of the Russian-Vietnamese Tropical Center (Ecolan 3.2 and Ecolan 1.2 projects). The morphological terminology follows [3,12]. The authors of the species and infraspecies names are mentioned in Table 2.

## 3. Results

**Figure 2:**
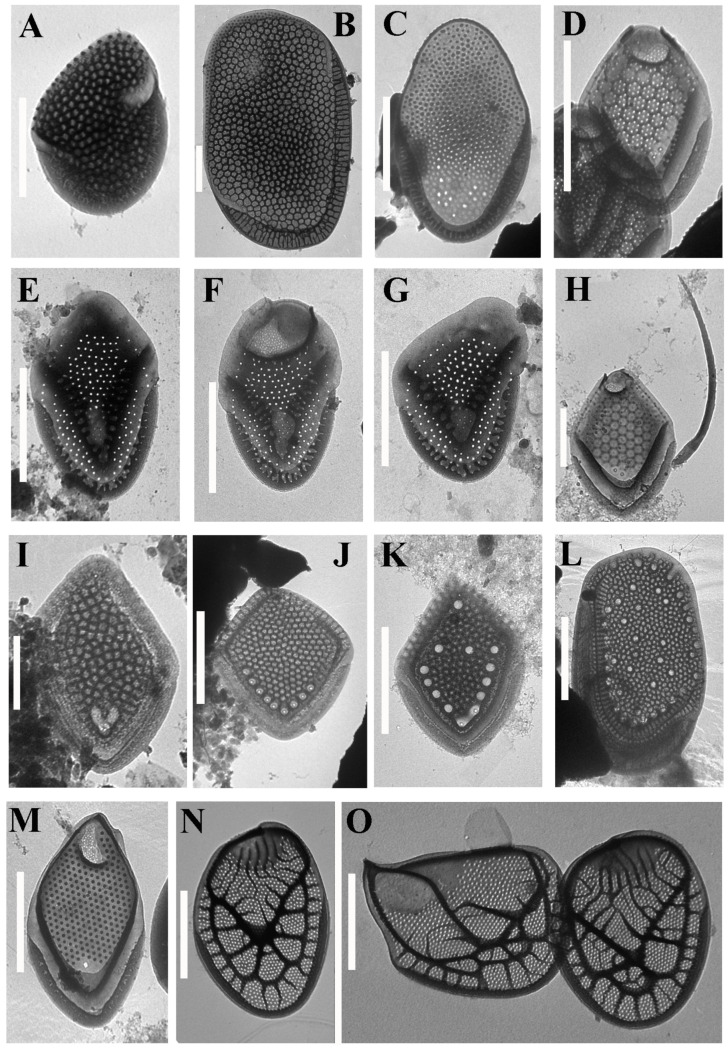
***Mallomonas* taxa from Phu Quoc and Con Son islands.** (**A**) *Mallomonas adamas*; (**B**) *Mallomonas bronchartiana*; (**C**) *Mallomonas ceylanica*; (**D**) *Mallomonas collucata*; (**E**–**G**) *Mallomonas cristata*, domeless body scale (**E**), domed body scale (**F**) and rear scale (**G**); (**H**) *Mallomonas distinguenda*; (**I**) *Mallomonas favosa*; (**J**) *Mallomonas favosa* f. *gemina*; (**K**) *Mallomonas* sp. 1; (**L**) *Mallomonas fimbriata*; (**M**) *Mallomonas furtiva*; (**N**,**O**) *Mallomonas harrisiae*, body scale (**N**), apical and body scales (**O**). Scale bars: (**A**–**G**,**J**–**O**): 2 μm; (**H**,**I**): 1 μm.

**Figure 3:**
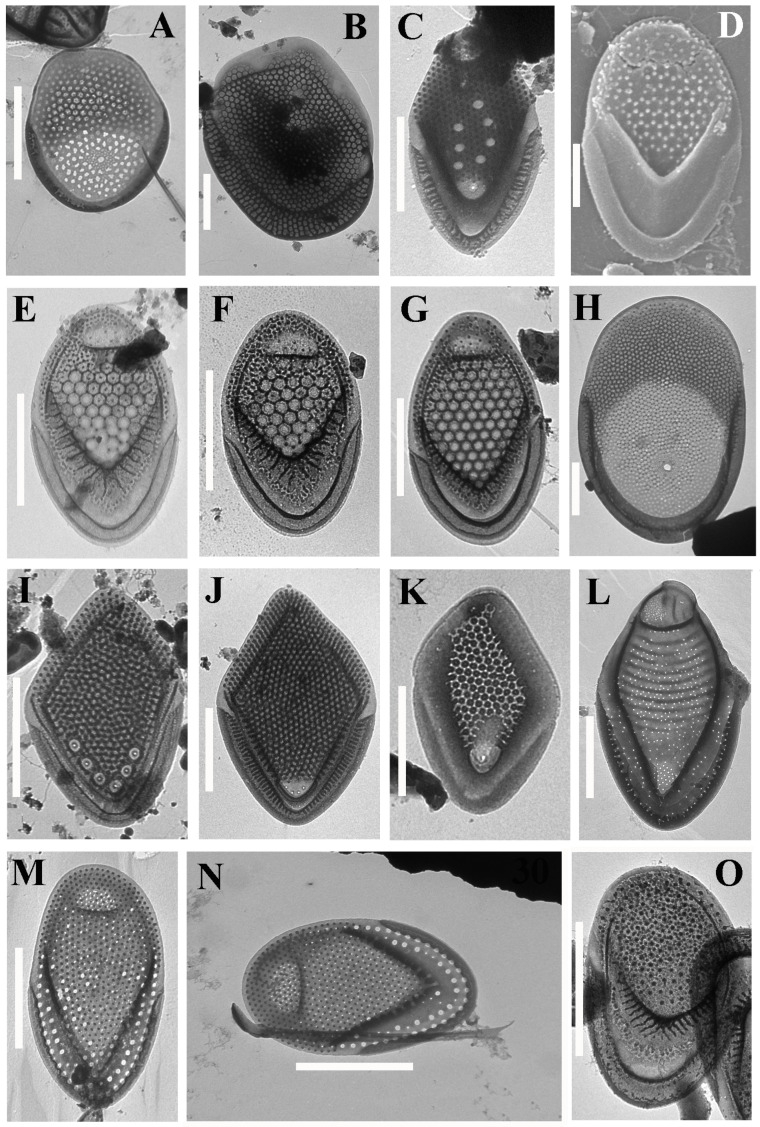
***Mallomonas* taxa from Phu Quoc and Con Son islands.** (**A**) *Mallomonas hexareticulata*; (**B**) *Mallomonas gusakovii*; (**C**) Mallomonas *guttata*; (**D**–**G**) *Mallomonas korshikovii*, SEM (**D**) and TEM (**E**–**G**); (**H**) *Mallomonas lamii*; (**I**) *Mallomonas mangofera* var. *foveata*; (**J**) Mallomonas *mangofera* var. *reticulata*; (**K**) *Mallomonas minuscula*; (**L**) *Mallomonas morrisonensis*; (**M**,**N**) *Mallomonas multisetigera*, scale (**M**), scale and bristle (**N**); (**O**) *Mallomonas* cf. *ouradion*. Scale bars: (**A**–**C**,**E**–**J**,**L**–**O**): 2 μm; (**D**,**K**): 1 μm.

**Figure 4:**
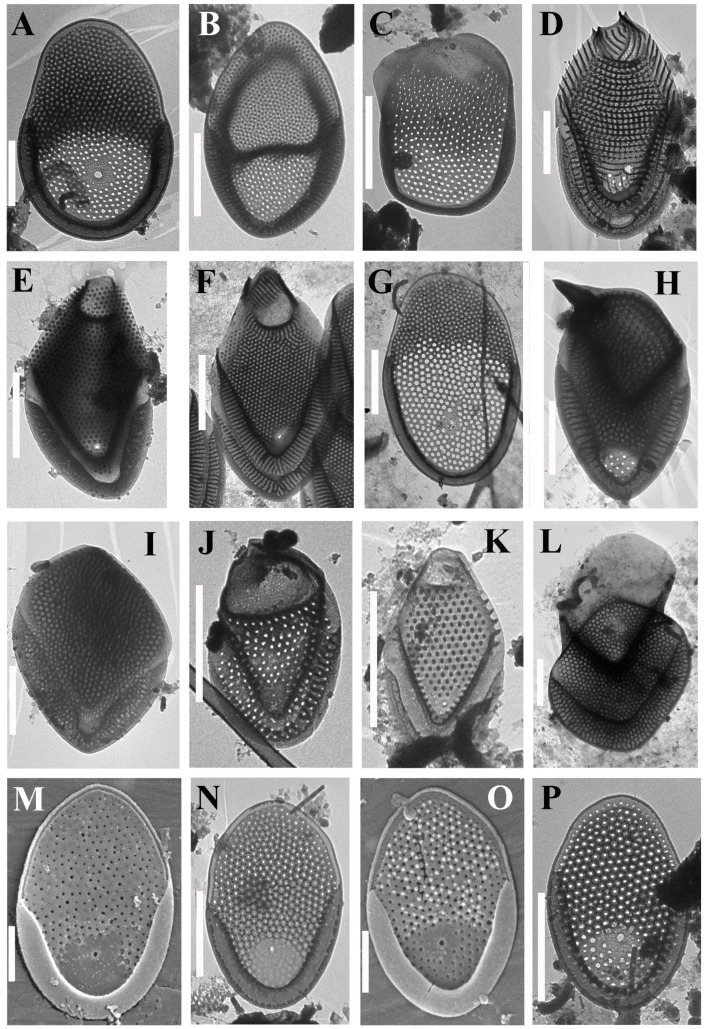
***Mallomonas* taxa from Phu Quoc and Con Son islands.** (**A**). *Mallomonas paragrandis*; (**B**). *Mallomonas peronoides*; (**C**). *Mallomonas plumosa*; (**D**) *Mallomonas punctostriata*; (**E**) *Mallomonas rasilis*; (**F**) *Mallomonas skvortsovii*; (**G**) *Mallomonas sorohexareticulata*; (**H**) *Mallomonas spinosa*; (**I**) *Mallomonas splendens*; (**J**) *Mallomonas tonsurata*; (**K**) *Mallomonas tropica*; (**L**) *Mallomonas velari*; (**M**,**N**) *Mallomonas pseudomatvienkoae*, SEM (**M**) and TEM (**N**); (**O**,**P**) *Mallomonas* sp. 2, SEM (**O**) and TEM (**P**). Scale bars: (**A**–**C**,**E**–**L**,**N**,**P**): 2 μm; (**D**,**M**,**O**): 1 μm.

**Figure 5:**
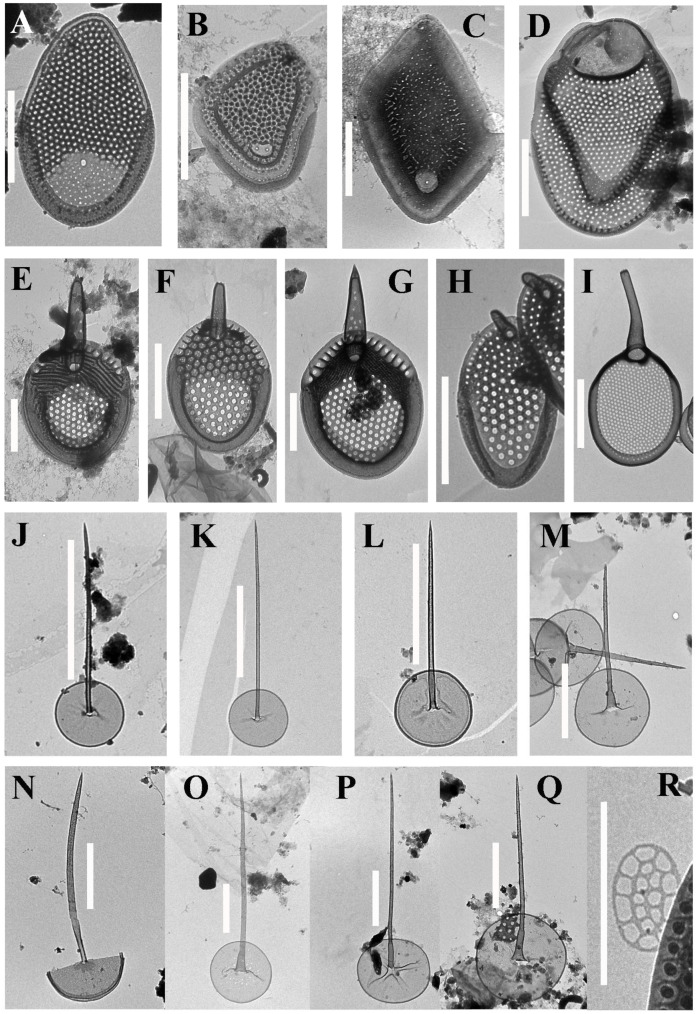
***Mallomonas, Synura, Paraphysomonas* and *Lepidochromonas* taxa from Phu Quoc and Con Son islands.** (**A**) *Mallomonas* sp. 3; (**B**) *Mallomonas* sp. 4; (**C**) *Mallomonas* sp. 5; (**D**) *Mallomonas* sp. 6; (**E**) *Synura echinulata*; (**F**) *Synura* cf. *longitubularis*; (**G**) *Synura mammillosa*; (**H**) *Synura papillosa*; (**I**) *Synura sphagnicola*; (**J**) *Paraphysomonas cambrispina*; (**K**) *Paraphysomonas longispina*; (**L**) *Paraphysomonas* cf. *variosa*; (**M**) *Paraphysomonas vulgaris*; (**N**) *Paraphysomonas* sp. 1; (**O**) *Paraphysomonas* sp. 2; (**P**) *Paraphysomonas* sp. 3; (**Q**) *Paraphysomonas* sp. 4; (**R**) *Lepidochromonas* sp. Scale bars: (**A**,**B**,**D**,**F**,**H**–**Q**): 2 μm; (**C**,**E**,**G**,**R**): 1 μm.

**Figure 6:**
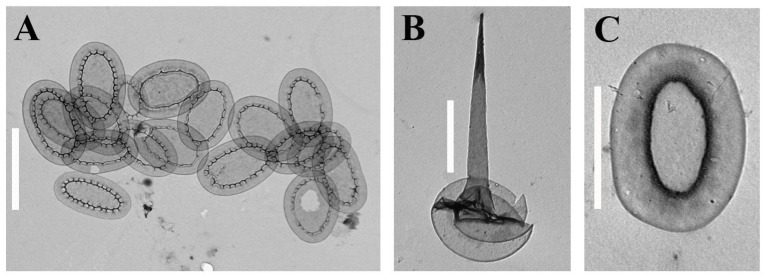
***Chrysosphaerella* and *Spiniferomonas* taxa from Phu Quoc Island.** (**A**,**B**) *Chrysosphaerella* sp., a group of plate-like scales (**A**) and a spine-like scale (**B**); (**C**) *Spiniferomonas* sp. Scale bars: (**A**,**B**): 2 μm; (**C**): 1 μm.

Altogether, 57 taxa were identified at 14 localities on the two islands (Table 2, Figure 2, Figure 3, Figure 4, Figure 5 and Figure 6). Forty-one taxa belong to the genus *Mallomonas*, while the others are species of the genera *Synura* (5), *Paraphysomonas* (8), *Spiniferomonas* (1), *Chrysosphaerella* (1) and *Lepidochromonas* (1). We report the genus *Lepidochromonas* for the first time in Viet Nam, and two species from the genus *Paraphysomonas* are also reported for the first time in the country. Six taxa from the genus *Mallomonas* and five taxa from the genus *Paraphysomonas* were not identified to the lower rank and may represent new species.

The studied localities on Phu Quoc Island had a pH less than 7 and low mineralization values, except for one swamp (Table 1). The only lake on Con Son Island for which parameters are available also had a pH less than 7 and low specific conductance values. In Phu Quoc Island, 44 taxa of silica-scaled chrysophytes were found, and 32 were found in Con Son Island. Nineteen species were common to the two regions studied (see Table 2), that is, only one-third of the taxa found. The most common were *Mallomonas mangofera* var. *foveata* and *M.* cf. *mangofera* var. *reticulata*, found in half of the localities. *Mallomonas favosa* f. *gemina* and *M. pseudomatvienkoae* were also usual taxa for both islands. The number of species in the samples varied very significantly, from 1 to 22. The highest species numbers were found in the swamp in the upper stream of the Duong Dong River on Phu Quoc Island (22) and in a water body at the site of a dried-up Quang Trung Reservoir (20) on Con Son Island. A quite high number of taxa (11–13) were observed in ponds near Quang Trung Reservoir (Con Son Island) and Duong Dong Reservoir (Phu Quoc Island).

Six unidentified morphotypes of the genus *Mallomonas* were found during our investigations. Presumably, these are new species for science, but their description requires more samples of scales of different types or the use of molecular methods.

***Mallomonas* sp. 1** (Figure 2K) belongs to the *Torquatae* section. The body scale is rhomboid and 3.5 × 2.5 µm in size. The shield is delimited by submarginal ribs (anterior and posterior) with arms of unequal length and reticulated with polygonal meshes. The anterior flanges have struts. The posterior border and flange are narrow. There are several rounded pits on the shield. Most of the pits are located along the posterior submarginal rib, and some are located in the central and distal parts of the scale. These pits do not contain rimmed pores in the center. Two rimmed pores are localized in the angle of the posterior submarginal rib. At the time of collection, the pH was 4.7, the temperature was 25 °C and the specific conductance was 29 μS cm^−1^.

The scale of *Mallomonas* sp. 1 is similar to those of *M. favosa* f. *favosa* and *M. favosa* f. *gemina*. *Mallomonas* sp. 1 is distinguished from these taxa by the presence of several pits without rimmed pores on the surface of the scale.

***Mallomonas* sp. 2** (Figure 4O,P) belongs to the *Planae* section. In this section, it is similar to taxa from the *M. matvienkoae* complex. It has oval scales that are 3.5–4.7 × 2.3–3.2 μm in size. Almost the entire area of the scale is covered with a secondary siliceous layer, which is thicker in the distal part of the scale and thinner in the proximal third, except for a small area in the center of the proximal part, where the secondary layer is absent. Papillae cover the shield, except for the proximal area with a thin secondary layer. There are a number of small pores along the anterior flange. The base-plate pores are distributed more or less evenly over the entire area of the scale. In the distal part, their diameter is slightly smaller than it is in the proximal part. The posterior border is wide, encircling half of the scale perimeter. The scales of *Mallomonas* sp. 2 are similar to the scales of *M.* sp. 3 in this study. However, *M.* sp. 3 has ovoid scales and an area without a secondary silica layer in the proximal part.

***Mallomonas*sp. 3** (Figure 5A) belongs to the *Planae* section. In this section, it is similar to taxa from the *M. matvienkoae* complex. It has ovoid scales that are 4.0–4.9 × 2.4–3.0 μm in size. Numerous small base-plate pores are distributed evenly on the distal two-thirds of the scale. A secondary reticulation with small rounded meshes covers the distal two-thirds of the scales. A large proximal pore is in the posterior portion of the scale without secondary reticulation. This morphotype was found previously in Central [42] and Northern Viet Nam [40]. *Mallomonas* sp. 3 was found with a pH range of 6.0–6.3, a specific conductance of 117 to 186 μS cm-1 and a temperature of 32–33 °C (Table 1).

The *Mallomonas matvienkoae* complex is a complicated group which needs the typification of *Mallomonas matvienkoae* Asmund & Kristiansen with molecular methods for its revision and the further description of new species. Thus, in this paper, we avoid describing these two new morphotypes as new species, although they certainly are.

***Mallomonas* sp. 4** (Figure 5B) belongs to the *Torquatae* section. Only one scale was found. The caudal scale is a wide rhomboid that is 2.9 × 2.4 µm in size. The anterior and posterior submarginal ribs are well defined. The shield and anterior flanges are covered with papillae. There are also a number of papillae on the posterior flange. There is a “window” without a secondary siliceous layer and with two rimmed pores in the angle of the posterior submarginal rib. Small base-plate pores are scattered along the shield, anterior and posterior flanges, with one row on the posterior flange along the posterior border and the second row along the posterior submarginal rib. Analogues with such an ultrastructure are not known. However, the discovery of only one caudal scale does not allow for a description of the new species. At the time of collection, the pH was 4.7, the temperature was 25 °C and the specific conductance was 29 μS cm^−1^.

***Mallomonas* sp. 5** (Figure 5C) belongs to the *Torquatae* section. The body scales are rhomboid, with distinct wide anterior flanges, 2.4–2.8 × 1.4–2.0 µm in size. The anterior and posterior submarginal ribs are well developed. The shield is covered with a secondary siliceous layer, forming a labyrinthine pattern of short ribs and gaps between them. At the angle of the posterior submarginal rib, there is an area without a secondary layer (“window”), which has a rounded shape with a rimmed pore in the center. The posterior border is narrow. The posterior flange is wide. At the time of collection, the pH was 4.7, the temperature was 25 °C and the specific conductance was 29 μS cm^−1^.

This species is somewhat similar to *M. madagascariensis* P. Hansen, *M. crocodilorum* P. Hansen [46], *M. minuscula* Gusev et al. [43] and *M. mangofera* f. *gracilis* Dürrschmidt [47]. However, the scales of the above-mentioned taxa are ornamented with papillae, which are absent in *Mallomonas* sp. 5. In *M. madagascariensis* and *M. mangofera* f. *gracilis*, papillae tend to fuse together. In contrast to *Mallomonas* sp. 5, *M. madagascariensis* has 3–4 pores in the “window” and *M. crocodilorum* has 1–3 pores, and the shape of the window in both species differs from that in *Mallomonas* sp. 5.

***Mallomonas* sp. 6** (Figure 5D) belongs to the *Mallomonas* section. The scales are oval, tripartite and 4.9–5.8 × 3.3–4.0 μm in size. The dome is large, rounded, oval and asymmetrical. The V-rib is acute and slightly hooded, and it is continuous with the arms of the anterior submarginal ribs. The shield has a secondary siliceous layer forming circular or elongated oval meshes, unevenly distributed on the surface. Numerous base-plate pores are placed irregularly. The posterior rim is narrow, and the posterior flange is wide. Similar scales are documented from other tropical regions, most often under the species epithet *Mallomonas corymbosa* Asmund & Hilliard [19,20,48]. Initially, *M. corymbosa* was described in Alaska [49] and was later determined to have a bipolar distribution [12]. However, the morphotype of *Mallomonas* sp. 6 differs from that of *M. corymbosa* in terms of the features of the development of the secondary silica layer on the shield and the wide anterior margins, especially on scales without a dome. The scales of *Mallomonas* sp. 6 are smaller than those of *M. corymbosa*. In Viet Nam, this morphotype was previously reported in the Mekong Delta under the name *Mallomonas* cf. *corymbosa* [39] and as *Mallomonas* sp. 2 in Northern Viet Nam [40], Central Viet Nam [42] and Southern Viet Nam [50].

In our studies, we also found several morphotypes similar to previously described taxa, but with some important differences. They require more research and more material to study. We provide descriptions here.

***M.* cf. *ouradion*** Harris & Bradley (Figure 3O). This is a fairly rare species with a scattered distribution [12], which was originally described in Great Britain [51]. However, our findings in Viet Nam differ from the scales recorded in the temperate zone. In contrast to the original description of *M. ouradion*, the scales from Phu Quoc Island are smaller (3.4–3.7 × 2.1–2.4 μm) and wide oval-shaped, with a wide posterior border, a V-rib shifted to the center of the scales, a poorly developed anterior submarginal rib and well-developed anterior flanges. Recently, another taxon similar in ultrastructure to *Mallomonas ouradion*, *M*. *cronbergiae* Piątek, was described in Africa (Cameroon) [52]. However, it differs from the type in having significantly smaller scales, a higher density of papillae on the surface of the scales and the presence of a number of pores on the posterior flange. The last 2 characters also distinguish *Mallomonas cronbergiae* from the morphotype from Viet Nam.

***Mallomonas* cf. *cristata*** Dürrschmidt (Figure 2E–G). *Mallomonas cristata* was first described in water bodies in Chile [53] and was later found in many habitats around the world [12]. In particular, in the tropics, the species is known in Madagascar [54], Brazil [32,55] and Ecuador [56]. Specimens from Viet Nam differ mainly in having serrated bristles, while in *M. cristata*, only 1–2 small teeth in the distal part of the bristles are indicated in the species diagnosis. The structure of the bristles is one of the characters for distinguishing species of the genus *Mallomonas*—in particular, in the sections *Mallomonas* and *Striatae* [57,58,59]. Most likely, the findings from Viet Nam represent a new species, but additional studies of the morphological structure and molecular data are needed to describe it.

***M. mangofera*** cf. var. ***reticulata*** (G. Cronberg) Kristiansen (Figure 3J) is an insufficiently described taxon which requires additional revision. However, it can be argued with certainty that its status should be raised to that of a separate species. It is believed that its distribution is limited to the tropics [12]. Initially, the scales of *Mallomonas mangofera* f. *reticulata* G. Cronberg were described in Zimbabwe. According to the description and holotype of Cronberg ([21], Figures 45–49), the scales have a triangular network, while the scales from Viet Nam have smaller round or polygonal meshes. The morphotype found in Viet Nam and the African morphotype also differ in terms of the shape of the scales, and they most likely should be considered different species based on morphological differences. However, we present our taxon as *M. mangofera* cf. var. *reticulata* due to the fact that the significance of such a feature as the form of internal reticulation on the scales is not yet clear, and further revision of this group is required. Moreover, the scales of this taxon from Viet Nam correspond to the scales defined under different epithets in other works devoted to the study of the flora of silica-scaled chrysophytes. In particular, scales identified as *Mallomonas mangofera* var. *reticulata* with polygonal or rounded cells are presented in studies from China ([20], Figures 56 and 57, p. 892; [60], Figure 47, p. 35). *Mallomonas mangofera* f. *reticulata*, with a similar structure, was recorded in Malaysia ([18], Figure 5, p. 253). Moreover, this morphotype was identified as *Mallomonas mangofera* var. *mangofera* from Jamaica ([21], Figure 44, p. 214), China ([61], Figure 46; [62], Figure 52) and Madagascar ([54], Figure 35, p. 161). Nemcova et al. ([63], Figures 29 and 30, p. 17) presented both morphotypes from French water bodies, with triangular ([63], Figure 30, p. 17) and polygonal ([63], Figure 29, p. 17) reticulation, under the name *Mallomonas mangofera* var. *reticulata*. To date, this morphotype, in addition to the regions mentioned above, is also known in India [16].

A diverse flora of the genus *Paraphysomonas* has also been revealed.

***Paraphysomonas cambrispina*** Scoble & Cavalier-Smith (Figure 5J). The scales of this species have a round basal-plate that is 0.9–1.0 µm in diameter, without a transverse crease and with a dense rim. The spine is 2.7–2.8 µm long, tapering to the oblique dull tip. This species has been described in an Austrian freshwater lake [3]. This is the first record from Viet Nam. However, despite the morphological similarity, the genetic identity of European and Vietnamese materials should be confirmed.

***Paraphysomonas longispina*** Scoble & Cavalier-Smith (Figure 5K). This species has one form of spine scales. The spines are 2.38–5.68 µm long, tapering to a dull point. The base-plate is round and 1.1–1.47 µm in diameter, with a dense rim. This species had previously been recorded in Mexico [3] and Viet Nam [39].

***Paraphysomonas*** cf. ***variosa*** Scoble & Cavalier-Smith (Figure 5L). Only a single scale has been observed. The base plate is round and 1.3 µm in diameter, with a dense rim. The spine is 3.11 µm long, tapering to an attenuated blunt tip. Our specimen differs from the type in having a denser rim. Another species with such a spine tip is *P. stylata* Scoble & Cavalier-Smith; however, its type subspecies, *P. stylata* subsp. *stylata*, is marine, and the freshwater subspecies *P. stylata* subsp. *limnetica* has a much longer spine (3.8–7.2 µm). This species had previously been recorded in India [3]. This is the first record of this species in Viet Nam.

***Paraphysomonas vulgaris*** subsp. ***vulgaris*** Scoble & Cavalier-Smith (Figure 5M). The scales of this taxon have a round basal-plate that is 2.02–2.04 µm in diameter, with a dense rim. The spine is 3.28–3.95 µm long, gently tapering from a wide bulbous base to an oblique dull pointed tip. This taxon has previously been recorded in the United Kingdom, Switzerland [3], Viet Nam [39] and Indonesia [64]. At the time of collection, the pH was 6.3, the temperature was 32 °C and the specific conductance was 186 μS cm^−1^.

***Paraphysomonas* sp. 1** (Figure 5N). Only a single scale has been observed. The base-plate (2.34 µm in diameter) is finely perforated and has a dense margin. The spine is 5.89 µm long, with an acute tip.

There are a few perforated *Paraphysomonas* species, e.g., *P. foraminifera* Lucas, *P. perforata* Scoble & Cavalier-Smith, *P. oligocycla* Takahashi, *P. porosa* Dürrschmidt & Cronberg, etc., but all of them have scales with base-plates smaller than 1 µm and larger perforations than those of our specimen. Therefore, our specimen represents a new, undescribed *Paraphysomonas* species.

***Paraphysomonas* sp. 2** (Figure 5O). This species has one type of spine scale. The spine is 3.27–8.91 µm long, gently tapering from a wide base to the tip; the spine base width is 0.24–0.58 µm. The round base-plate with a thickened margin is 2.15–3.14 µm in size. The base-plate is irregularly perforated, mainly in the central area. This species differs from all other *Paraphysomonas* species in terms of scale and spine dimensions and in having scattered irregular perforations of the base-plate. Remarkably, extremely similar scales were reported by Wujek & Saha [65] regarding Indian material. It is unknown whether such irregular perforations have taxonomic value, or if they are caused by problems in silicification, but it is very likely that our scales represent currently undescribed species.

***Paraphysomonas* sp. 3** (Figure 5P). Only a single scale has been observed. The scale base-plate of this species has four radial creases. The only taxon that has such radial creases is *Paraphysomonas uniformis* subsp. *hemiradia* Scoble & Cavalier-Smith. However, the scale of our specimen is significantly larger (2.65 µm vs. 1.5–2 µm in *P. uniformis* subsp. *hemiradia*) and has a longer spine (7.2 µm vs. 3.6–5.8 µm in *P. uniformis* subsp. *hemiradia*). So, we cannot unambiguously identify our specimen as *P. uniformis* subsp. *hemiradia*. Very similar scales were reported in Indonesian Papua ([64], Figure 2B,C). At the time of collection, the pH was 5.0, the temperature was 31 °C and the specific conductance was 1200 μS cm^−1^.

***Paraphysomonas* sp. 4** (Figure 5Q). Only two scales have been observed. The base plate is round, 2.78–2.82 µm in diameter, with a dense rim. The spine 4.85–5.7 µm long, tapering from the bulbous base to the oblique dull pointed tip. The S/P ratio is 1.7–2.1. Our scales resemble those of *P. vulgaris* subsp. *vulgaris* Scoble & Cavalier-Smith but are much larger (2.78–2.82 µm vs. 1.8–2.2 µm in *P. vulgaris* subsp. *vulgaris*) and have a longer spine (4.85–5.7 µm vs. 3.1–4.5 µm in *P. vulgaris* subsp. *vulgaris*). It is very likely that our scales represent an undescribed *Paraphysomonas* species. At the time of collection, the pH was 6.3, the temperature was 32 °C and the specific conductance was 186 μS cm^−1^.

***Lepidochromonas* sp.** (Figure 5R). Only a single plate scale has been observed. The plate scale (0.59 × 0.38 µm) bears 10 holes in the outer ring and 8 holes in the central area. The plate scales of *Lepidochromonas caronii* (Scoble & Cavalier-Smith) Kapustin & Guiry have a similar structure, but this species is marine, whereas our specimen originates from freshwaters. In any case, the observation of all scale types is required for correct identification. At the time of collection, the pH was 6.3, the temperature was 32 °C and the specific conductance was 186 μS cm^−1^.

## 4. Discussion

In total, 57 taxa of silica-scaled chrysophytes were recorded from 14 localities on two small tropical islands. This is an exceptionally high species richness. For instance, in mainland Malaysia, only 30 taxa have been registered [13]. During the investigation of more than 200 Indian waterbodies, 58 taxa of silica-scaled chrysophytes have been reported [15,16,65]. Studies of 11 Chinese provinces located in the tropical and subtropical parts of the country revealed only 49 taxa of silica-scaled chrysophytes [19,20]. In other tropical countries (e.g., Brazil, Colombia, Guatemala and Jamaica), the number of taxa is less than 30 [21,32]. Interestingly, some areas in the temperate zone with comparable diversity to that in our study have been recognized as biodiversity hot spots for silica-scaled chrysophytes, e.g., the Aquitaine region in France, where 58 taxa of *Synura* and *Mallomonas* were recorded [63], and the Bolshezemelskaya tundra, with 75 taxa of silica-scaled chrysophytes [66].

The data on the biodiversity of silica-scales chrysophytes from islands are rather limited. Dürrschmidt & Cronberg [17] identified 29 taxa from Sri Lanka, including two new species (*Mallomonas ceylanica* Dürrschmidt & Cronberg, *Paraphysomonas porosa* Dürrschmidt & Cronberg) and one new variety (*Mallomonas matvienkoae* var. *grandis* Dürrschmidt & Cronberg). Couté & Franceschini [55] recorded 20 species from Santa Catarina Island (Brazil). In Madagascar, 42 taxa have been recorded [46,54,67], including four new species: *Mallomonas madagascariensis* Hansen, *M. lemuriocellata* Hansen, *M. crocodilorum* Hansen and *Spiniferomonas cetrata* Hansen. Kim et al. [68] identified 25 species of *Mallomonas* and *Synura* and recorded 4 unidentified *Mallomonas* species from Jeju Island (Korea). Later, several new *Mallomonas* species from Jeju Island were described, e.g., *M. jejuensis* H.S. Kim & J.H. Kim [69] and *M. elevata* H.S. Kim [70]. Wei et al. [20] found 36 taxa of silica-scaled chrysophytes from Hainan Island (China) and described a new species, *Paraphysomonas hainanensis* Wei & Kristiansen, which, however, does not fit *Paraphysomonas* sensu stricto. We studied the diversity of silica-scaled chrysophytes in the island of Java and identified only 19 taxa, including the new species *Chrysosphaerella nichollsii* Kapustin & Gusev [71]. In Indonesian Papua, we recorded 24 taxa, including a “living fossil”, *Mallomonas preisigii* Siver [64], and described a new species, *Mallomonas papuensis* Kapustin, Gusev & Kulikovskiy [72]. Interestingly, both species were discovered in bog pools in the highlands [64,72]. In the neighboring area of Papua New Guinea, Vyverman & Cronberg [73] identified only 20 taxa, without reporting any novel taxa. This brief review shows that almost every taxonomic study of silica-scaled chrysophytes from tropical islands has resulted in the description of new species. All of the taxa mentioned above, described in the islands, remain endemic, except for *Mallomonas matvienkoae* var. *grandis*, which is found in tropical and subtropical regions in South and North America, Asia and Africa [12]. However, the findings of this taxon need to be revised.

In addition to the species richness, the flora of the silica-scaled chrysophytes of the two studied islands is characterized by endemic species which have so far only been noted in Viet Nam or neighboring countries of Southeast Asia. The strict endemics of Phu Quoc are *Mallomonas collucata* (Figure 2D) and *Mallomonas gusakovii* (Figure 3B). The first species belongs to the *Annulatae* section and is known in only one site [45]. The second species belongs to the *Quadratae* section and was described from two swamps of the island [44]. *Mallomonas punctostriata* (Figure 4D), *M. velari* (Figure 4L), *M. skvortsovii* (Figure 4F) and *M. distinguenda* (Figure 2H) are endemics of Viet Nam and are very rare taxa known only in a limited number of localities [45,74,75]. The first two taxa were discovered in our study for the second time after the initial description. *Mallomonas distinguenda* was reported in six more habitats in Central and Southern Viet Nam [42,50]. *Mallomonas minuscula* and *M. lamii* are the other endemics of Viet Nam, but they are widespread in the country and can most likely be found in neighboring countries [43,76]. In this study, we present the second locality of the recently described *Mallomonas fimbriata* [77]. Previously, a scale similar to *Mallomonas fimbriata* was found in Southern Viet Nam (Binh Thuan Province); however, it has some morphological differences and may be a different taxon [50]. The scales of this taxon were also found in Malaysia as *Mallomonas* sp. 3 ([13], Figure 49, p. 294). *Mallomonas korshikovii* was first found and described based on SEM studies in swamp pools with high mineralization on the site of mangroves on the Cam Ranh Peninsula [36]. Subsequently, it was found in five more localities in Southern and Central Viet Nam [42,50]. Here, we present several TEM images of this very rare taxon (see Figure 3E–G). Three more species described in Viet Nam, *Mallomonas cattiensis*, *M. spinosa* Gusev and *M. furtiva*, with a wide distribution in the country [37,40,78,79,80], have already been found elsewhere—the first in Papua New Guinea [73] (identified as *M. morrisonensis*) and Indonesia [71], the second in China [20] and Indonesia [71] and the third in the Indonesian part of Papua New Guinea [64] and Malaysia [13].

Tropical islands often possess species-rich and highly endemic algal florae. For example, in New Caledonia, Moser et al. [81] recorded 643 diatom taxa, 257 of which were considered endemic. The authors even christened New Caledonia as the “Galapagos of diatoms” [81] because of the exceptional quantity of endemic diatoms. Metzeltin & Lange-Bertalot [82] observed 287 taxa of diatoms in Madagascar, including 43 species new to science and 177 endemic species. Sherwood [83], who compiled the first checklist of non-marine algae of the Hawaiian islands, noted the surprisingly low level of endemism, at only 5%. However, more recent studies suggested that the number of endemics may be underestimated [84,85,86,87]. Floristic and taxonomic studies of algae from Indonesia have revealed a great diversity in particular groups, e.g., diatoms [88,89,90,91,92,93,94,95,96,97] and desmids [98,99,100,101], and have resulted in descriptions of many generic- and specific-level endemics.

The very diverse flora of the chrysophytes of Phu Quoc and Con Son islands is similar to that of the swamp areas of the mainland of Viet Nam, such as the wetlands in the Cat Tien National Park and the coastal swamps of Cam Ranh Peninsula and Thua Thien Hue Province [37,38,42]. The number of such habitats is very small and is constantly declining due to agricultural and tourism activities. Water bodies of different types on the islands, where human economic activity is not yet as highly developed as it is on the mainland, serve as reserves of rare species. Thus, as in the case of higher plants and animals, even small islands support a very rich taxonomic diversity of microalgae that includes many endemic or rare species as well as taxa new to science. This illustrates the need to protect natural freshwater habitats and to conduct a detailed inventory of other groups of protists, which is necessary to identify the full diversity and conservation of endemic species.

## Figures and Tables

**Table 1:** Basic characteristics of the studied localities (Cond.—specific conductance, µS/cm, T—temperature, °C).

No.	Locality	Coordinates	pH	Cond.	T
**Phú Quốc Island**					
1	Duong Dong Reservoir	N 10°15.039′ E 104°1.279′	5.7	6	31
2	Pond near Doung Dong Reservoir	N 10°14.937′ E 104°1.300′	6.1	13	32
3	Swamp 1 in the upper stream of Duong Dong river	N 10°13.908′ E 103°59.114′	4.7	25	29
4	Swamp 2 near the estuary of Cua Can river	N 10°19.152′ E 103°52.958′	5.0	1200	31
5	Swamp 3 near the estuary of Cua Can river	N 10°19.174′ E 103°52.856′	4.6	24	27
6	Pond 1 in the northwest part	N 10°19.856′ E 103°51.538′	6.0	117	33
7	Pond 2 in the northwest part	N 10°19.834′ E 103°51.537′	6.3	186	32
**Côn Sơn Island**					
8	Quang Trung Reservoir 1	N 8°41.516′ E 106°36.364′	n/a	n/a	n/a
9	Quang Trung Reservoir 2	N 8°41.528′ E 106°36.372′	n/a	n/a	n/a
10	Aquaculture pond 1 near Quang Trung Reservoir	N 8°41.540′ E 106°36.363′	n/a	n/a	n/a
11	Aquaculture pond 2 near Quang Trung Reservoir	N 8°41.573′ E 106°36.350′	n/a	n/a	n/a
12	Aquaculture pond 3 near Quang Trung Reservoir	N 8°41.558′ E 106°36.332′	n/a	n/a	n/a
13	Aquaculture pond 4 near Quang Trung Reservoir	N 8°41.550′ E 106°36.323′	n/a	n/a	n/a
14	Lake Ahn Hai	N 8°40.645′ E 106°35.867′	6.5	201	30

**Table 2:** List of silica-scaled chrysophytes found in Phu Quoc and Con Son islands (new taxa for Viet Nam are given in bold; “+” indicates the presence of taxon; “–”—absence).

Taxon	1	2	3	4	5	6	7	8	9	10	11	12	13	14
Synurales														
*Mallomonas adamas* K.Harris & D.E.Bradley	**+**	**–**	**–**	**–**	**–**	**–**	**–**	**–**	**–**	**–**	**–**	**–**	**–**	**–**
*Mallomonas bronchartiana* Compère	**–**	**–**	**–**	**–**	**–**	**–**	**–**	**+**	**–**	**–**	**–**	**–**	**+**	**+**
*Mallomonas ceylanica* Dürrschmidt & Cronberg	**–**	**–**	**–**	**–**	**–**	**–**	**–**	**–**	**+**	**–**	**–**	**+**	**+**	**–**
*Mallomonas collucata* Gusev & Kulikovskiy	**–**	**–**	**+**	**–**	**–**	**–**	**–**	**–**	**–**	**–**	**–**	**–**	**–**	**–**
*Mallomonas* cf. *cristata* Dürrschmidt	**–**	**+**	**–**	**–**	**–**	**–**	**–**	**–**	**–**	**–**	**–**	**–**	**–**	**–**
*Mallomonas distinguenda* Gusev et al.	**–**	**–**	**–**	**–**	**+**	**–**	**–**	**–**	**–**	**–**	**–**	**–**	**–**	**–**
*Mallomonas favosa* Nicholls	**+**	**+**	**+**	**–**	**–**	**–**	**–**	**+**	**–**	**–**	**–**	**–**	**–**	**–**
*Mallomonas favosa* f. *gemina* Dürrschmidt & Croome	**–**	**–**	**+**	**–**	**–**	**–**	**+**	**+**	**+**	**–**	**–**	**+**	**+**	**–**
*Mallomonas* sp. 1	**–**	**–**	**+**	**–**	**–**	**–**	**–**	**–**	**–**	**–**	**–**	**–**	**–**	**–**
*Mallomonas fimbriata* Gusev	**–**	**–**	**+**	**–**	**–**	**–**	**–**	**–**	**–**	**–**	**–**	**–**	**–**	**–**
*Mallomonas furtiva* Gusev, Certnerová, Škaloudová & Škaloud	**–**	**+**	**–**	**–**	**–**	**+**	**–**	**–**	**–**	**–**	**–**	**–**	**–**	**–**
*Mallomonas gusakovii* Gusev, Kapustin, Martynenko, Guseva & Kulikovskiy	**–**	**–**	**+**	**+**	**–**	**–**	**–**	**–**	**–**	**–**	**–**	**–**	**–**	**–**
*Mallomonas harrisiae* Takahashi	**+**	**–**	**–**	**–**	**–**	**–**	**–**	**–**	**–**	**–**	**–**	**–**	**–**	**–**
*Mallomonas hexareticulata* B.Y.Jo, W.Shin, H.S.Kim, P.A.Siver & R.A.Andersen	**–**	**–**	**–**	**–**	**–**	**+**	**–**	**–**	**–**	**–**	**–**	**–**	**–**	**–**
*Mallomonas guttata* Wujek	**–**	**+**	**+**	**–**	**–**	**–**	**–**	**–**	**+**	**–**	**–**	**+**	**–**	**–**
*Mallomonas korshikovii* Gusev	**–**	**–**	**–**	**+**	**–**	**–**	**–**	**+**	**–**	**–**	**–**	**–**	**–**	**+**
*Mallomonas lamii* Gusev, Kulizin, Guseva, Shkurina & Kulikovskiy	**–**	**–**	**–**	**–**	**–**	**–**	**–**	**+**	**–**	**–**	**–**	**–**	**–**	**+**
*Mallomonas mangofera* var. *foveata* (Dürrschmidt) Kristiansen	**–**	**–**	**+**	**–**	**–**	**+**	**+**	**+**	**+**	**–**	**–**	**+**	**+**	**–**
*Mallomonas mangofera* var. *reticulata* (Cronberg) Kristiansen	**–**	**–**	**–**	**–**	**–**	**+**	**+**	**+**	**–**	**–**	**+**	**+**	**+**	**+**
*Mallomonas minuscula* Gusev, Guseva, Kezlya & Kulikovskiy	**–**	**–**	**–**	**–**	**–**	**+**	**+**	**–**	**+**	**–**	**–**	**–**	**–**	**–**
*Mallomonas morrisonensis* Croome & P.A. Tyler	**–**	**–**	**–**	**–**	**–**	**+**	**+**	**–**	**–**	**–**	**–**	**–**	**–**	**–**
*Mallomonas multisetigera* Dürrschmidt	**–**	**–**	**+**	**–**	**–**	**–**	**–**	**+**	**–**	**–**	**–**	**–**	**–**	**–**
*Mallomonas* cf. *ouradion* Harris & Bradley	**–**	**–**	**+**	**–**	**–**	**–**	**–**	**–**	**–**	**–**	**–**	**–**	**–**	**–**
*Mallomonas paragrandis* Gusev	**–**	**+**	**–**	**–**	**–**	**–**	**–**	**+**	**–**	**+**	**–**	**–**	**–**	**+**
*Mallomonas peronoides* (K. Harris) Momeu & L.S. Péterfi	**–**	**–**	**–**	**–**	**–**	**–**	**–**	**+**	**+**	**–**	**–**	**+**	**+**	**–**
*Mallomonas plumosa* Croome & P.A. Tyler	**–**	**–**	**–**	**–**	**–**	**–**	**–**	**+**	**–**	**–**	**–**	**–**	**–**	**–**
*Mallomonas punctostriata* Gusev & Kulikovskiy	**–**	**–**	**–**	**+**	**–**	**–**	**–**	**–**	**–**	**–**	**–**	**–**	**–**	**–**
*Mallomonas rasilis* Dürrschmidt	**–**	**–**	**+**	**–**	**–**	**–**	**–**	**+**	**+**	**–**	**–**	**–**	**–**	**–**
*Mallomonas skvortsovii* Gusev et al.	**–**	**–**	**+**	**–**	**–**	**–**	**–**	**–**	**–**	**–**	**–**	**–**	**–**	**–**
*Mallomonas sorohexareticulata* Jo, Shin, Kim, Siver & Andersen	**–**	**+**	**+**	**–**	**–**	**–**	**–**	**–**	**–**	**–**	**–**	**–**	**–**	**–**
*Mallomonas spinosa* Gusev emend. Wei & Kristiansen	**–**	**–**	**+**	**–**	**–**	**–**	**–**	**–**	**–**	**–**	**–**	**+**	**+**	**+**
*Mallomonas splendens* (G.S. West) Playfair	**–**	**+**	**+**	**+**	**–**	**–**	**–**	**+**	**–**	**–**	**–**	**–**	**–**	**–**
*Mallomonas tonsurata* Teiling	**–**	**+**	**–**	**–**	**–**	**–**	**–**	**+**	**–**	**–**	**–**	**–**	**–**	**–**
*Mallomonas tropica* Dürrschmidt & Croome	**–**	**–**	**–**	**–**	**–**	**–**	**–**	**+**	**–**	**–**	**–**	**–**	**–**	**–**
*Mallomonas velari* Gusev, Siver & W. Shin	**–**	**–**	**+**	**–**	**–**	**–**	**–**	**–**	**–**	**–**	**–**	**–**	**–**	**–**
*Mallomonas pseudomatvienkoae* Jo, Shin, Kim, Siver & Andersen	**+**	**+**	**+**	**–**	**–**	**–**	**–**	**+**	**–**	**–**	**–**	**+**	**–**	**+**
*Mallomonas* sp. 2	**–**	**–**	**–**	**–**	**–**	**–**	**–**	**+**	**–**	**–**	**–**	**–**	**–**	**–**
*Mallomonas* sp. 3	**–**	**–**	**–**	**–**	**–**	**+**	**+**	**–**	**+**	**–**	**–**	**+**	**+**	**–**
*Mallomonas* sp. 4	**–**	**–**	**+**	**–**	**–**	**–**	**–**	**–**	**–**	**–**	**–**	**–**	**–**	**–**
*Mallomonas* sp. 5	**–**	**–**	**+**	**–**	**–**	**–**	**–**	**–**	**–**	**–**	**–**	**–**	**–**	**–**
*Mallomonas* sp. 6	**–**	**–**	**–**	**–**	**–**	**–**	**–**	**–**	**–**	**–**	**–**	**–**	**+**	**–**
*Synura echinulata* Korshikov	**–**	**–**	**+**	**–**	**–**	**–**	**–**	**–**	**–**	**–**	**–**	**–**	**+**	**–**
*Synura longitubularis* B.Y. Jo, W. Shin, J.I. Kim & Siver	**–**	**+**	**–**	**–**	**–**	**–**	**–**	**+**	**+**	**–**	**–**	**–**	**+**	**–**
*Synura mammillosa* Takahashi	**–**	**–**	**–**	**+**	**–**	**–**	**–**	**–**	**–**	**–**	**–**	**–**	**–**	**–**
*Synura papillosa* Kapustin, Gusev & Siver	**–**	**–**	**+**	**–**	**–**	**+**	**–**	**–**	**–**	**–**	**–**	**+**	**–**	**–**
*Synura sphagnicola* (Korshikov) Korshikov	**–**	**–**	**+**	**–**	**–**	**–**	**–**	**–**	**–**	**–**	**–**	**–**	**–**	**–**
Paraphysomonadales														
*Lepidochromonas* sp.	**–**	**–**	**–**	**–**	**–**	**–**	**+**	**–**	**–**	**–**	**–**	**–**	**–**	**–**
*Paraphysomonas cambrispina* Scoble & Cavalier-Smith	**–**	**–**	**–**	**–**	**–**	**–**	**–**	**–**	**–**	**–**	**–**	**–**	**–**	**+**
*Paraphysomonas longispina* Scoble & Cavalier-Smith	**–**	**–**	**–**	**–**	**–**	**–**	**–**	**–**	**–**	**–**	**–**	**–**	**+**	**+**
*Paraphysomonas* cf. *variosa* Scoble & Cavalier-Smith	**–**	**–**	**–**	**–**	**–**	**–**	**–**	**–**	**–**	**–**	**–**	**–**	**+**	**–**
*Paraphysomonas vulgaris* subsp. *vulgaris* Scoble & Cavalier-Smith	**–**	**–**	**–**	**–**	**–**	**–**	**+**	**–**	**–**	**–**	**–**	**–**	**–**	**–**
*Paraphysomonas* sp. 1	**–**	**–**	**–**	**–**	**–**	**–**	**–**	**+**	**–**	**–**	**–**	**–**	**–**	**–**
*Paraphysomonas* sp. 2	**–**	**–**	**–**	**–**	**–**	**–**	**–**	**–**	**–**	**–**	**–**	**+**	**–**	**–**
*Paraphysomonas* sp. 3	**–**	**–**	**–**	**+**	**–**	**–**	**–**	**–**	**–**	**–**	**–**	**–**	**–**	**–**
*Paraphysomonas* sp. 4	**–**	**–**	**–**	**–**	**–**	**–**	**+**	**–**	**–**	**–**	**–**	**–**	**–**	**–**
Chromulinales														
*Chrysosphaerella* sp.	**+**	**–**	**–**	**–**	**–**	**–**	**–**	**–**	**–**	**–**	**–**	**–**	**–**	**–**
*Spiniferomonas* sp.	**+**	**+**	**–**	**–**	**–**	**–**	**–**	**+**	**–**	**–**	**–**	**–**	**–**	**–**

## Data Availability

Not applicable.
